# An Engineered Separator with N-Doped Graphene Nanosheets for Trapping Polysulfides in Advanced Li-S Batteries

**DOI:** 10.3390/molecules31071172

**Published:** 2026-04-01

**Authors:** Bing Chen, Yiwen Li, Chaojiang Fan, Qingpei Zhou, Wenhu Li, Hang Su, Cong Li, Shixiong Zhang, Chenhui Yang, Teng Wang

**Affiliations:** 1 School of Materials Science and Engineering, Shaanxi University of Technology, Hanzhong 723001, China; chenbing@snut.edu.cn (B.C.); fanchaojiang@snut.edu.cn (C.F.);; 2 School of Chemistry and Chemical Engineering, Northwestern Polytechnical University, Xi’an 710072, China

**Keywords:** nitrogen-doped graphene nanosheets, lithium-sulfur batteries, sulfur, engineered separator, shuttle effect

## Abstract

Lithium–sulfur (Li-S) battery technology has attracted significant research interest owing to sulfur’s remarkable theoretical capacity and exceptional energy density potential. Nevertheless, the low conductivity of sulfur and the “shuttle effect” pose challenges to its practical applications. To enhance electrochemical performance, this work developed nitrogen-doped graphene (NG) nanosheets as a separator coating for Li-S battery. As a modification layer for separators, NG acts as a physical barrier that prevents polysulfides from migrating across the separator to reach the anode, thereby mitigating the shuttle effect. Additionally, NG improves the conductivity of the separator and enhances wettability between the separator and electrolyte, facilitating uniform transmission of lithium ions. Notably, NG functionalized separators demonstrate excellent mechanical flexibility, contributing to improved cycle stability for batteries. Furthermore, theoretical calculations indicate a strong interaction between NG and lithium polysulfides (LiPSs), effectively inhibiting polysulfide migration. The Li-S battery utilizing the NG modified separator maintains a capacity retention rate of 51.5% after 100 cycles at 0.1 C with a sulfur loading of 1.47 mg/cm^2^ and exhibits a capacity decay rate of only 0.092% after 500 cycles at a discharge rate of 1 C. This work highlights the potential advantages of employing NG as a separator coating layer in enhancing the electrochemical performance of the Li-S battery.

## 1. Introduction

The exponential growth of electronic devices and electric mobility solutions has created critical demand for advanced battery systems featuring superior energy storage capabilities and extended operational lifespans [1,2]. Modern energy storage technologies represented by conventional lithium-ion systems, which depend on reversible insertion/extraction processes of lithium ions for charge storage, face fundamental limitations in meeting contemporary requirements for high-performance power storage [3,4]. This technological gap has accelerated global research efforts toward developing novel secondary battery architectures with breakthrough energy densities. Lithium–sulfur electrochemical systems have emerged as a frontrunner in next-gen energy storage technologies, boasting unparalleled theoretical metrics, including a remarkable 1675 mAh/g specific capacity and an exceptional 2600 Wh/kg energy density [5,6,7]. Additional competitive advantages of Li-S battery technology encompass cost-effectiveness in material sourcing and reduced environmental impact compared to traditional battery chemistries [8,9,10,11]. Despite their significant promise, Li-S battery systems face major obstacles in practical implementation: (i) sulfur cathodes and their reduction products demonstrate poor electrical conductivity [12,13,14], while (ii) intermediate polysulfide species tend to undergo undesirable migration between electrodes during charge–discharge cycles [15,16,17,18]. These limitations collectively contribute to inefficient active material usage, compromised cycle life, and reduced charge retention capabilities, posing substantial barriers to widespread commercial adoption of this energy storage technology [19,20,21].

To address these issues, scientists have explored multiple approaches such as integrating sulfur-based cathodes with specialized functional components [22,23], creating innovative binding agents [24,25], and engineering sophisticated separator configurations [11,26,27]. Within lithium–sulfur battery systems, separators serve not only as physical barriers between electrodes but also exert a critical influence on controlling electrochemical processes and boosting cell efficiency. Contemporary studies focus on optimizing separator properties to suppress polysulfide migration, enhance charge transfer capabilities, and prolong operational durability, which has become a key focus in contemporary battery research.

Contemporary research identifies four principal material categories for enhancing polypropylene separators in energy storage systems: (i) carbonaceous substances [28], (ii) inorganic metallic components encompassing oxides [29], nitrides [30], carbides [31], and sulfides [32,33], (iii) polymeric compounds [34], and (iv) porous coordination polymers [35,36]. Over the past decade, carbon-based substances have emerged as particularly advantageous for separator optimization owing to their superior electrical conductivity, extensive surface areas, exceptional chemical durability, and tunable porous architectures [37]. Such characteristics not only boost charge transfer kinetics and ionic mobility but also effectively mitigate polysulfide migration through spatial restriction and surface interactions. These combined effects substantially elevate both the energy storage capabilities and operational longevity of Li-S cells. Nevertheless, it is important to note that different types of carbon materials possess distinct characteristics when utilized for separator modification. For instance, carbon nanotubes (CNTs) [38] demonstrate remarkable electrical conductivity and mechanical robustness, yet their tendency to form entangled clusters creates dispersion challenges in solvent-based applications. This particle agglomeration negatively affects coating consistency and functional performance. In contrast, carbon nanofibers (CNFs) [39] randomly stack to create open porous channels that enhance electrolyte wettability and facilitate rapid ion transport. Nevertheless, CNFs possess relatively lower conductivity and a limited specific surface area. Consequently, the rational selection of carbon materials and the achievement of efficient and stable performance through structural design and functional modification remain critical challenges in contemporary Li-S battery research. NG has become a research hotspot for the modification layer of lithium–sulfur (Li-S) battery separators due to its tunable electronic structure and excellent electrical conductivity. However, there is still a lack of systematic understanding of the dominant role of nitrogen doping configurations (especially pyrrolic nitrogen) in interface regulation and the intrinsic mechanism of their synergistic effect with the macroscopic morphology of the material.

In this study, a simple and efficient hydrothermal process was adopted to synthesize NG materials with significantly enriched pyrrolic nitrogen content and a large-area continuous network composed of closely packed and interconnected nanosheets, using urea and graphene oxide as precursors. This method has the advantages of high cost-effectiveness and suitability for large-scale production. When this NG material was used to modify the Celgard 2500 separator, the engineered separator could not only effectively block the shuttling of lithium polysulfides (LiPSs) but also improve the interfacial conductivity and charge transfer efficiency. Thanks to the synergistic effect of the chemical anchoring of pyrrolic nitrogen and the physical barrier/ion transport channel of the nanosheet network, the battery prototype with NG-modified separator exhibited excellent electrochemical performance: an initial discharge capacity of 1081.4 mAh/g at a 0.1 C rate, and a capacity of 312 mAh/g after 500 cycles at a 1 C rate, with a capacity decay rate of only 0.092% per cycle. It also demonstrated outstanding rate performance and significantly reduced self-discharge behavior.

## 2. Results and Discussion

**Figure 1:**
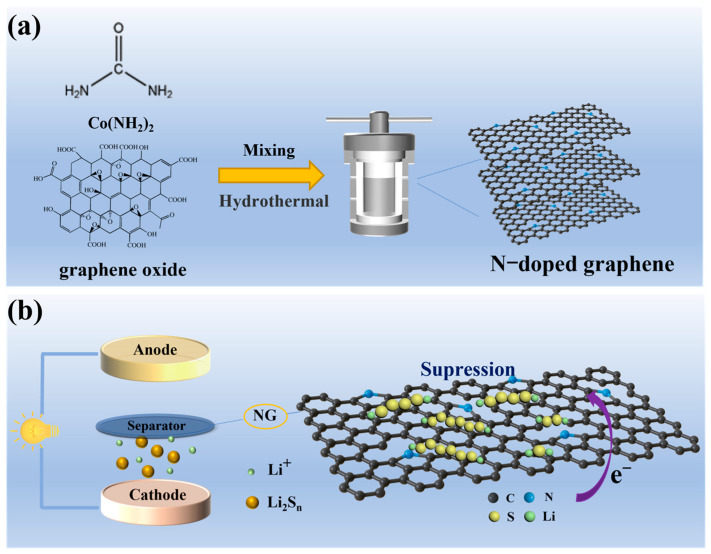
(**a**) Schematic illustration of the fabrication process of NG; (**b**) the operating mechanisms of NG/Celgard 2500 separator.

Figure 1a depicts the fabrication process of nitrogen-doped graphene material. The NG nanosheets were fabricated through a hydrothermal synthesis approach, utilizing graphene oxide combined with urea as the nitrogen precursor. Figure 1b depicts the operational mechanism of the NG-enhanced separators. This advanced composite separator demonstrates substantial performance enhancements compared to traditional polypropylene separators, featuring a thin NG modification layer applied to the cathode-contacting surface of the Celgard 2500 substrate. Throughout electrochemical cycling operations, the optimized electrical conductivity of the NG/Celgard 2500 hybrid separator promotes accelerated lithium-ion migration while simultaneously minimizing ohmic polarization effects. The conductive architecture further stimulates surface-mediated polysulfide redox activities, boosting active material usage rates and serving as an efficient secondary charge transfer interface. Furthermore, the incorporated NG component regulates electrolyte permeation patterns and refines ionic conduction networks, strengthening electrochemical reaction kinetics throughout battery operation.

**Figure 2:**
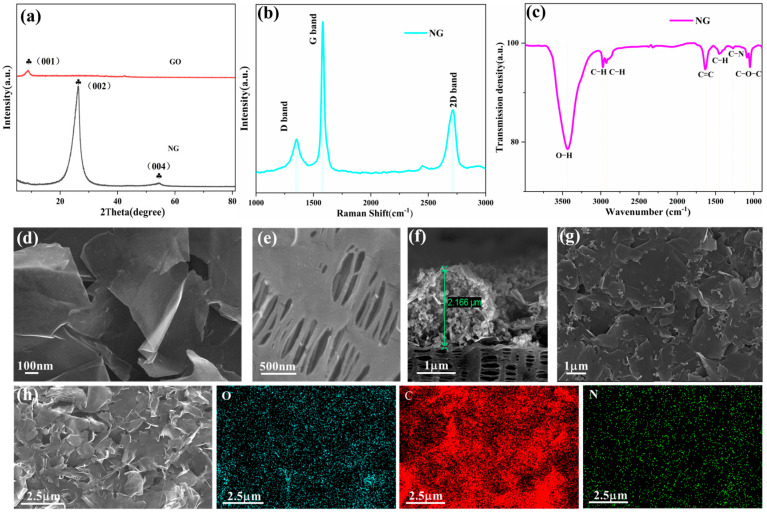
(**a**) XRD pattern of GO and NG; (**b**) Raman spectra of NG materials; (**c**) FTIR spectrometer of NG materials; (**d**) FESEM images of NG; (**e**) FESEM image of Celgard 2500 separator; (**f**) cross-sectional FESEM image of the NG/Celgard 2500 separator; (**g**) FESEM image of the surface of NG/Celgard 2500 separator; and (**h**) elemental distribution map of NG material.

As depicted in Figure 2a, the XRD pattern of graphene oxide displays a distinctive peak at 10°, which is attributed to the (001) crystallographic plane. For the NG material, a diffraction peak appearing at approximately 54.26° originates from the (004) plane, whereas the reflection centered at 26.32° arises from the (002) plane structure. Structural characterization through Raman spectroscopy (Figure 2b) reveals three distinct vibrational modes in the NG material. Three characteristic vibrational bands are identified at 1352 cm^−1^ (D-band), 1581 cm^−1^ (G-band), and 2714 cm^−1^ (2D-band). The presence of the G band signifies that the material possesses a graphite-like structure, whereas the D-band intensity reflects structural imperfections and lattice disorder. The intensity ratio of the D band to that of the G band (I_D_/I_G_) serves as a crucial indicator of defect content within graphene. For NG specimens, the calculated I_D_/I_G_ ratio reaches 0.27, while the I_2D_/I_G_ value stands at 0.45, suggesting that NG materials consist of multilayer graphene. Fourier-transform infrared spectroscopic analysis (Figure 2c) revealed characteristic absorption bands corresponding to various molecular vibrations. A distinct absorption band at 3434 cm^−1^ represents O-H bond stretching, while dual peaks at 2972 cm^−1^ and 2927 cm^−1^ originate from C-H stretching modes. The spectral feature at 1633 cm^−1^ demonstrates aromatic C=C ring vibrations, complemented by a 1451 cm^−1^ peak indicative of C-H deformation. Critical evidence of nitrogen incorporation appears as a 1271 cm^−1^ signal corresponding to C-N stretching, with additional confirmation from 1087 cm^−1^ and 1048 cm^−1^ absorptions characteristic of C-O-C stretching vibrations.

Field emission scanning electron microscopy (FESEM) characterization reveals the NG material’s architecture as extensive, stacked, and interlinked nanosheets across broad regions, as depicted in Figure 2d. Elemental composition analysis through EDX spectroscopy (Figure 2h) demonstrates successful nitrogen incorporation within the carbon matrix, along with uniform dispersion of C, N, and O elements throughout the material. The NG surface displays a predominantly defect-free morphology characterized by gentle undulations along sheet peripheries. Comparative FESEM examination of the microporous Celgard 2500 separator (Figure 2e) exposes an extensive network of submicron-sized conduits exceeding hundreds of nanometers in diameter. These interconnected channels serve as unrestricted pathways for polysulfide migration between electrodes. Furthermore, the separator’s limited compatibility with polar electrolytes results in incomplete pore wetting, as evidenced by contact angle measurements. A thin layer measuring approximately 2.16 μm in thickness was deposited onto the microporous Celgard 2500 separator’s surface (Figure 2f). It can also be seen from the surface of the diaphragm in Figure 2g that it is uniformly covered with a layer of NG material. This surface modification demonstrates significant effectiveness in suppressing polysulfide migration.

**Figure 3:**
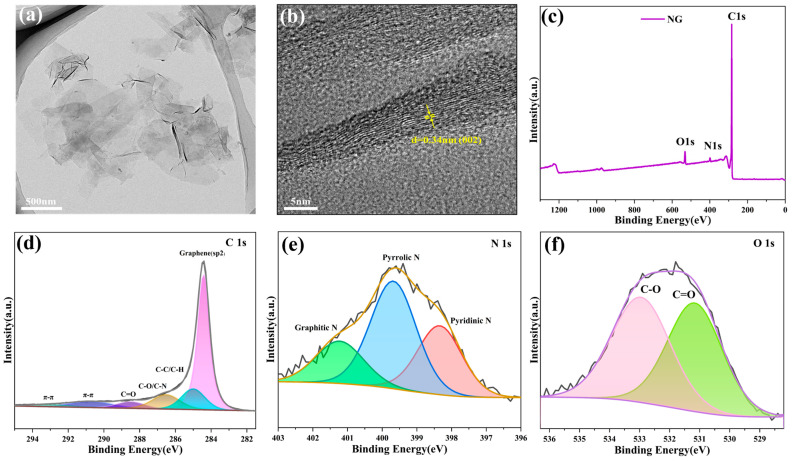
(**a**) TEM image and size distribution of NG; (**b**) HRTEM image of NG; (**c**–**f**) XPS spectra of NG: (**c**) XPS survey spectrum of NG; (**d**) high-resolution C 1s spectrum of NG; (**e**) high-resolution N1s spectrum of NG; and (**f**) high-resolution O 1s spectrum of NG.

The structural characteristics and size distribution of NG were examined using transmission electron microscopy (TEM). Figure 3a presents a typical TEM micrograph captured from a planar observation angle. The NG graphene sheets exhibit a thin, translucent structure, demonstrating that the synthesized material adopts a nano-sheet configuration devoid of significant aggregation. As shown in Figure 3b, high-resolution TEM analysis of NG demonstrates clear lattice fringes, with an interplanar distance measuring 0.34 nm, aligning with the characteristic (002) crystallographic plane of graphite [40]. For a comprehensive evaluation of elemental constituents and chemical bonding configurations in NG samples, X-ray photoelectron spectroscopy (XPS) analysis was performed. This surface-sensitive analytical technique generates photoelectron emissions exclusively from the material’s outermost atomic layers. The XPS spectral data for NG specimens, displayed in Figure 3c, demonstrates two prominent spectral signatures centered at binding energies of approximately 284.78 eV and 401.00 eV, corresponding to C 1s and N 1s signals, respectively [41], verifying effective nitrogen incorporation within the graphene framework. The C 1s spectrum of NG can be deconvoluted into six distinct forms of carbon: characteristic peaks at 284.4 eV (graphene sp^2^), 285.0 eV (C-C/C-H bonds), 286.5 eV (C-O/C-N bonds), 288.5 eV (carbonyl groups), 290.7 eV, and 293.1 eV (π-π bonds). Oxygen species analysis in Figure 3f demonstrates two primary oxygen states at 531.0 eV (C=O) and 533.0 eV (C-O) [42]. High-resolution N1s spectrum analysis (Figure 3e) identifies three nitrogen configurations, pyridinic N (395.6 eV), pyrrolic N (400.0 eV), and graphitic N (401.8 eV) [43], with pyrrolic nitrogen constituting the dominant species. The presence of edge-nitrogen configurations (pyridinic and pyrrolic) exhibits superior catalytic activity compared to graphitic nitrogen, substantially improving the material’s charge transport efficiency and electrochemical performance through enhanced surface reactivity and optimized electron transfer pathways.

**Figure 4:**
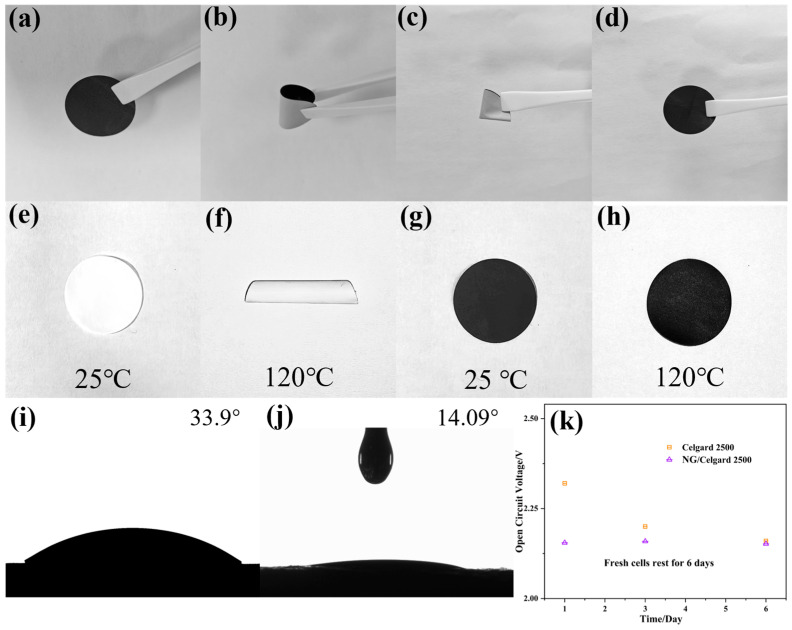
(**a**–**d**) NG/Celgard 2500 separator after bending and recovery; (**e**–**h**) Heat treatment diagrams of Celgard 2500 and NG/Celgard 2500 separators; (**i**) The contact angles of Celgard 2500 separators with electrolyte; (**j**) The contact angles of NG/Celgard 2500 separators with electrolyte; (**k**) OCV profiles showing self-discharge behavior.

The NG material was applied onto the Celgard 2500 separator, as illustrated in Figure 4a–d. The modified separator demonstrated excellent flexibility through bidirectional bending and folding tests without any delamination of NG particles from the functionalized surface. Remarkably, the altered separator maintained complete structural recovery after deformation cycles with no observable fractures, confirming its exceptional mechanical durability. High-temperature stability represents a crucial parameter for separator performance in high-performance battery applications. When subjected to 120 °C thermal exposure, the Celgard 2500 separator underwent substantial shrinkage and warping, as shown in Figure 4e–h, while the NG/Celgard 2500 composite separator preserved its original dimensions with negligible distortion. These findings verify the NG/Celgard 2500 separator’s enhanced structural integrity under thermal stress, significantly improving battery safety during thermal runaway scenarios. The electrolyte wettability characteristics were further investigated through contact angle measurements. The electrolyte wettability of Celgard 2500 and the modified NG/Celgard 2500 separator was assessed through contact angle measurements. Experimental data illustrated in Figure 4i showed that the baseline Celgard 2500 exhibited a contact angle of 33.9°, while the NG-incorporated counterpart demonstrated notably lower hydrophobicity with a 14.09° measurement (Figure 4j). This substantial improvement in surface compatibility stems from oxygen-rich functional groups within the NG coating, which significantly boosts electrolyte interaction capabilities. Such enhanced interfacial properties enable optimized electrolyte distribution across the separator matrix, thereby improving lithium-ion mobility while potentially minimizing electrode–electrolyte interfacial impedance. Comparative analysis of self-discharge characteristics (Figure 4k) through open-circuit voltage monitoring revealed stable electrochemical performance in NG/Celgard 2500-equipped cells during six-day resting periods. The superior electrolyte-philic nature of the composite separator emerges as a critical factor in maintaining voltage stability during inactive states. Over the testing period, the open-circuit voltage of NG/Celgard 2500 maintained stability at 2.152 V. This voltage decline in Li-S batteries’ self-discharge mechanism mainly stems from redox-induced potential reduction. The elemental sulfur interacts with dissolved lithium ions to generate intermediate polysulfide compounds, which subsequently diffuse across the separator and undergo reactions with metallic lithium. Experimental OCV analysis reveals that nitrogen-doped graphene demonstrates significant effectiveness in inhibiting the migration of polysulfide species.

**Figure 5:**
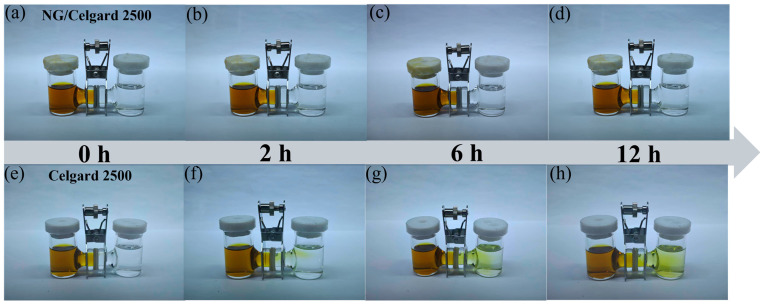
(**a**–**d**) Photos of Li_2_S_6_ penetration tests in H-type glass cells with NG/Celgard 2500 separator; and (**e**–**h**) Celgard 2500 separator.

To evaluate the blocking capability of NG separators toward polysulfide migration, Li_2_S_6_ diffusion experiments were performed in an H-type glass apparatus, as illustrated in Figure 5. Experimental findings revealed that solutions separated by a conventional separator exhibited yellow discoloration within 12 h, visually confirming substantial polysulfide crossover. Conversely, when employing the NG-modified separator, the receiving chamber maintained transparency without observable color changes throughout the testing period. This observation confirms the NG layer’s effectiveness in suppressing polysulfide migration, consequently mitigating the detrimental shuttle phenomenon in battery systems.

**Figure 6:**
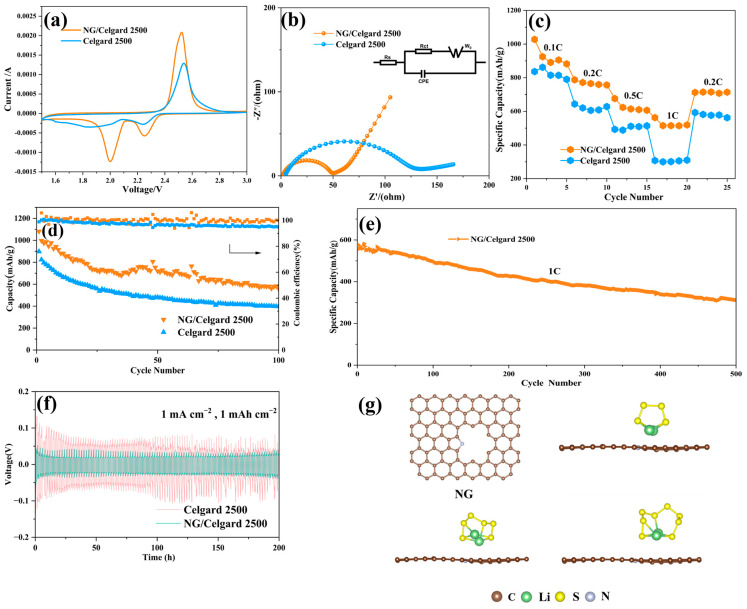
(**a**) CV profiles with NG/Celgard 2500 and Celgard 2500 at 0.1 mV/s; (**b**) The EIS curves with NG/Celgard 2500 and Celgard 2500; (**c**) Rate performance of NG/Celgard 2500 and Celgard 2500; (**d**) Charging/discharging profiles under 0.1 C of the NG/Celgard 2500 and Celgard 2500; (**e**) Long-term cycling durability of the NG/Celgard 2500 separator at 1 C; (**f**) The cycling performance of Li-Li symmetrical cells assembled with NG/Celgard 2500 and Celgard 2500 separators at a current density of 1 mA cm^−2^; (**g**) The configuration and binding energy of sulfur species in pyrrolic nitrogen-doped graphene.

Electrochemical characterization via cyclic voltammetry (CV) was performed to evaluate the battery’s performance. Figure 6a displays the CV profiles of the NG/Celgard 2500 separator recorded between 1.5 and 3.0 V potential window. The Li-S cells employing the NG/Celgard 2500 separator reveal two well-defined reduction peaks near 2.05 V and 2.30 V, corresponding to the sequential sulfur reduction mechanism. The higher potential peak at 2.30 V represents sulfur conversion to long-chain lithium polysulfides (Li_2_S_x_, 4 ≤ x ≤ 8), while the lower 2.05 V peak signifies subsequent reduction to short-chain Li_2_S_2_/Li_2_S products [44]. Oxidation peaks, conversely, reflect the reverse sulfur regeneration process. When contrasted with conventional Celgard 2500 separators, the NG-modified variant demonstrates intensified redox peak intensities, indicative of enhanced electrochemical activity and improved reaction kinetics.

As illustrated in Figure 6b, the electrochemical impedance spectroscopy (EIS) analysis compares the performance of Li-S batteries utilizing NG/Celgard 2500 and conventional Celgard 2500 separator. The semicircle in the high-frequency region corresponds to the charge transfer resistance (*R*_ct_), and the straight line in the low-frequency region represents the Warburg impedance, which is related to the diffusion rate of Li^+^ [45,46]. The results indicate that the *R*_ct_ of the NG/Celgard 2500 separator is lower than that of the Celgard 2500 separator, suggesting that the former is more conducive to promoting charge transfer.

Figure 6c presents comparative rate capability evaluations for Li-S cells employing NG/Celgard 2500 and conventional Celgard 2500 separators. Benefiting from enhanced electrical conductivity, the NG/Celgard 2500 configured cell demonstrates capacities of 1027.8 mAh/g (0.1 C), 787.5 mAh/g (0.2 C), 675.9 mAh/g (0.5 C), and 562.4 mAh/g (1 C). Upon returning to a 0.2 C rate, the system maintains 712.5 mAh/g capacity, indicating favorable reversibility. Comparatively, Celgard 2500 separator-based cells exhibit significantly lower performance metrics across all tested current densities, with particularly pronounced capacity degradation at elevated rates. The material demonstrates an initial discharge capacity of 836.4 mAh/g under 0.1 C, progressively dropping to 643.3 mAh/g at 0.2 C. This performance continues diminishing to approximately 493.0 mAh/g under 0.5 C conditions, ultimately reaching 307.3 mAh/g when subjected to 1 C current density. Comparative cycling stability evaluations between separator configurations, as depicted in Figure 6d, reveal distinct performance characteristics. The NG/Celgard 2500 separator-equipped cell achieves an initial capacity of 1081.4 mAh/g at a rate of 0.1 C, retaining 557.6 mAh/g after 100 cycles with 51.5% capacity preservation while maintaining coulombic efficiencies exceeding 99% throughout testing. Conversely, Celgard 2500 separator cells demonstrate inferior performance metrics, commencing with 897.5 mAh/g initial capacity that deteriorates to 396.7 mAh/g post-cycling, corresponding to 44.2% capacity retention and reduced 95% coulombic efficiency. Long-term electrochemical behavior analysis presented in Figure 6e further illustrates the NG-coated separator’s enhanced stability characteristics. Under 1 C current density conditions during 500-cycle testing, Li-S cells equipped with NG-modified separators exhibited a remarkable initial capacity of 578.5 mAh/g. After 500 charge–discharge cycles, these cells maintained a residual discharge capacity of 312.0 mAh/g, corresponding to an exceptionally low average capacity loss per cycle of merely 0.092%. This electrochemical stability highlights the material’s superior cycling durability. The experimental data confirms that NG-enhanced separators substantially improve battery longevity and capacity preservation through two primary mechanisms: The modified separator creates an efficient physical blockade against polysulfide crossover while simultaneously providing chemical anchoring points. Surface functional groups in NG materials, particularly pyridinic and pyrrolic nitrogen species, enable strong chemisorption interactions that effectively suppress polysulfide dissolution and migration.

To examine the modulatory influence of the NG separator on lithium-ion deposition patterns and their consequences for anode stability, a Li-Li symmetric cell system was utilized to evaluate the long-term cycling performance. As depicted in Figure 6f, under consistent current conditions of 1 mA cm^−2^, the NG-modified cell sustained an overpotential of approximately 30 mV throughout 200 operational hours. This performance metric notably surpasses that of the unmodified control cell. These experimental outcomes confirm the NG-enhanced separator’s capability to orchestrate homogeneous lithium-ion distribution during both deposition and stripping processes, thereby markedly inhibiting dendritic growth patterns and improving electrochemical interface durability at the metallic lithium electrode.

To investigate the interactions between nitrogen-doped graphene and polysulfides, density functional theory (DFT) simulations were performed to analyze polysulfide adsorption characteristics. The study focused on three representative lithium polysulfide species: Li_2_S_4_, Li_2_S_6_, and Li_2_S_8_. Computational models simulating pyrrolic nitrogen-doped graphene’s interaction with these polysulfides were developed, followed by binding energy calculations. Figure 6g reveals adsorption energies of −0.88 eV, −0.64 eV, and −0.52 eV for Li_2_S_4_, Li_2_S_6_, and Li_2_S_8_, respectively, when interacting with pyrrolic nitrogen-doped graphene.

## 3. Experimental Section

### 3.1. Synthesis of N-Doped Graphene

The N-doped graphene synthesis involved introducing 50 mg of graphene oxide (15–20 pieces, 4–10% edge oxidation, Aladdin, Shanghai, China) into 20 mL of deionized water, followed by the incorporation of 1 g of urea (AR, ≥99%, Aladdin). After 30 min sonication, the homogeneous dispersion was loaded into a 50 mL autoclave and thermally treated at 180 °C for 24 h. The hydrothermal reactor was subsequently naturally cooled to ambient temperature, yielding the final N-doped graphene product with enhanced structural stability.

### 3.2. Preparation of NG/Celgard 2500 Separator

NG, polyvinylidene fluoride (PVDF), and Super P Li were mixed in a mass ratio of 8:1:1 and homogenized uniformly for 7 h using a magnetic stirrer to prepare the coating slurry. The resulting slurry was applied onto the surface of a commercial polypropylene separator (Celgard 2500, USA Celgard, Charlotte, NC, USA) via doctor-blade coating. The coated separator was then dried under vacuum at 60 °C for 12 h to remove residual solvent. Following drying, the separator was punched into circular discs with a diameter of 19 mm. During battery assembly, the NG-coated side of the separator was oriented toward the sulfur cathode.

### 3.3. Preparation of Sulfur Cathode and Battery Assembly

The sulfur cathode was fabricated via a slurry-coating technique. A composite material formulated with 70% elemental sulfur, 20% conductive carbon (Super P), and 10% PVDF binder was initially dispersed in N-methylpyrrolidone solvent. This homogeneous mixture underwent six hours of mechanical agitation to achieve optimal viscosity. The prepared slurry was subsequently blade-coated onto aluminum current collectors and subjected to overnight vacuum drying at 60 °C. For the PP separator-based cells, the sulfur loading reached 1.0 mg/cm^2^, whereas the NG separator counterparts exhibited higher loadings of 1.4–1.5 mg/cm^2^. CR2032 (Kelude New Energy Technology Co., Ltd., Shenzhen, China) coin cells were assembled in an argon-filled glove box to eliminate moisture and oxygen contamination. Metallic lithium foil functioned as the counter electrode. A pure lithium foil served as the anode, while either the original polypropylene (PP) separator or the NG-PP separator separated the electrodes. The liquid electrolyte consisted of 1 M LiTFSI dissolved in a mixed solvent system, with 2% lithium nitrate additive incorporated to enhance electrochemical stability. In this electrolyte formulation, the volume ratio of 1,3-dioxolane (DOL) to 1,2-dimethoxyethane (DME) is 1:1. Following cell assembly, the batteries underwent a 12 h resting period to ensure complete electrolyte saturation (60 μL) before electrochemical evaluation.

### 3.4. Lithium Symmetric Cell Configuration

Symmetric cells were fabricated under an inert atmosphere using lithium metal foils as dual-functioning electrodes. The electrode pair was separated using either unmodified polypropylene (PP) membranes or NG-PP separators. The electrolyte formulation consisted of 1 M lithium bis (trifluoromethanesulfonyl)imide (LiTFSI) dissolved in DOL/DME (1:1 *v*/*v*) with 2 wt.% lithium nitrate additives.

### 3.5. Material Characterization

The morphological characteristics of the NG specimen were examined through field emission scanning electron microscopy (FESEM, Zeiss Supra 55, Carl Zeiss, Oberkochen, Germany). Contact angle measurements for the separator were conducted using a goniometric system (CA, JC2000D1, Shanghai Zhongchen Digital Technology Equipment Co., Ltd., Shanghai, China). Structural features at the nanoscale were studied with transmission electron microscopy (TEM, FEI Tecnai G2 Spirit, Now part of Thermo Fisher Scientific, Brno, Czech Republic). Raman spectroscopic analysis (Raman, Horiba LabRAM HR800, Horiba Jobin Yvon S.A.S, Palaiseau, France) was applied to assess chemical bonding features. Surface elemental composition and oxidation states were determined via X-ray photoelectron spectroscopy (XPS, Thermo Fisher ESCALAB 250Xi, Thermo Fisher Scientific, Waltham, MA, USA). The molecular structure of NG composites was characterized using Fourier-transform infrared spectroscopy (FTIR, Nicolet iS 10, Thermo Fisher Scientific, Waltham, MA, USA).

### 3.6. Electrochemical Measurements

Electrochemical characterization was conducted through cyclic voltammetry experiments using a CHI600E electrochemical workstation (Shanghai Chenhua Instrument Co., Ltd., Shanghai, China), applying a scanning rate of 0.1 mV/s across a potential window spanning 1.5–3.0 V. Electrochemical impedance spectroscopy analysis was performed using the identical instrument, with measurements recorded over a frequency spectrum of 100 kHz to 10 mHz. Battery cycling stability and rate capability assessments were executed under ambient conditions employing a Land CT3002A battery cycler (Wuhan Landian Electronics Co., Ltd., Wuhan, China), with the operating voltage range strictly controlled within 1.5–3.0 V throughout testing.

### 3.7. Theoretical Calculations

All of the calculations are performed in the framework of the spin-polarized density functional theory with the projector augmented plane-wave method, as implemented in the Vienna ab initio simulation package (VASP 5.4). The Perdew Burke-Ernzerh of (PBE) functional of the Generalized Gradient Approximation (GGA) method was employed to describe the electron exchange–correlation interactions. The ions–electrons interaction was described using the projection enhanced wave method (PAW). The cutoff energy was set to 500 eV in all calculations. The Monkhorst Pack k-mesh of 3 × 2 × 1 was used for structural optimization. During the structural relaxation process, the convergence criteria for residual force and energy were set to 0.03 eV/Å and 10^−5^ eV, respectively. A vacuum slab of 15 Å was employed to separate periodic images along the c direction. The adsorption energy of Li_2_S_x_ was defined as E_ads_ = E_total_ − E_slab_ – E_adsorbate_, where E_total_ is the total energy of the adsorbate adsorbed on the substrate material, E_slab_ denotes the energy of the substrate material, and E_adsorbate_ is the energy of the adsorbate. 

## 4. Conclusions

In summary, NG nanosheets were effectively fabricated through a controlled synthesis process and implemented as a versatile surface modification for standard Celgard 2500 separators in lithium–sulfur energy storage systems. The engineered NG interface demonstrates dual functionality as both an enhanced charge transfer medium and a durable shield against polysulfide migration. The material’s superior electrolyte affinity, evidenced by reduced wetting angles, facilitates rapid ionic conduction while minimizing ohmic losses. The separator maintains excellent structural integrity at high temperatures, and its safety performance is also enhanced. Remarkably, the modified separator maintains minimal self-discharge (6-day idle period) and enables cells to achieve unprecedented cycling durability, sustaining 1 C operation for 500 cycles with only 0.092% capacity degradation per cycle, showcasing exceptional high-rate electrochemical stability conditions. This study proposes a novel approach for developing and engineering multifunctional anchoring-catalytic separator systems in next-generation Li-S battery systems.

## Data Availability

The original contributions of this study are included in this article. For any further questions, please contact the corresponding author.
